# Editorial: Differential Efficacy of Immune Checkpoint Inhibitors due to Age and Sex Factors

**DOI:** 10.3389/fimmu.2022.941254

**Published:** 2022-06-16

**Authors:** Pei Pei Chong, Joshua B. Rubin, Maha Abdullah, Fabio Conforti, Sabra L. Klein, Wanli Liu

**Affiliations:** ^1^ School of Biosciences, Faculty of Health and Medical Sciences, Taylor’s University, Subang Jaya, Malaysia; ^2^ School of Medicine, Washington University at St. Louis, St. Louis, MO, United States; ^3^ Department of Pathology, Faculty of Medicine and Health Sciences, Universiti Putra Malaysia, Serdang, Malaysia; ^4^ Division of Medical Oncology for Melanoma, Sarcoma & Rare Tumours, European Institute of Oncology (IEO), Milan, Italy; ^5^ W Harry Feinstone Department of Molecular Microbiology and Immunology, Johns Hopkins University, Baltimore, MD, United States; ^6^ School of Life Sciences, Institute for Immunology, Ministry of Education (MOE) Key Laboratory of Protein Sciences, Beijing Advanced Innovation Center for Structural Biology, Collaborative Innovation Center for Diagnosis and Treatment of Infectious Diseases, Beijing Key Lab for Immunological Research on Chronic Diseases, Tsinghua University, Beijing, China; ^7^ Tsinghua-Peking Center for Life Sciences, Beijing, China

**Keywords:** immune checkpoint inhibitors, age and sex, cancer immunotherapy, age and sex-based different immune responses, PD-1/PD-L1, anti-CTLA-4

Immune checkpoint inhibitors (ICI) represented by PD-1/PD-L1 inhibitors and anti-CTLA-4 agents have increasingly become a prominent modality for cancer therapy, while the potential of ICI for the treatment of chronic infections is only beginning to be intensively assessed. The heterogeneous and complex nature of cancers make ICI favorable to be developed as a personalized medicine to maximize its efficacy and suit individual needs. Indeed, it is now widely accepted that not every patient benefits from ICI as inadequate response rates, complications from immune-related adverse events, toxicity, and increased risk of infection drastically limit the efficacy of ICI. Increasing evidence shows that some of these failures are due to immune variations from aging as well as genetic differences such as sex. The aim of this Research Topic, including both original research articles and review articles, is to highlight the current understanding of how age and sex factors influence the efficacy of ICI in cancer immunotherapy.

The immune status of an individual measured by routine tests including differential white blood counts or lymphocyte subpopulation enumeration is unlikely to show differences between male and female. The state of immune response is best observed when activated. *In vitro* mitogen stimulation demonstrated significant differences in response of natural killer cells and T cells between male and females (1). Thus, results from ICI trials which showcase direct targeting of immune components are extraordinary opportunities to demonstrate intrinsic workings of the immune system and offer insights into sex and age influences.

Sex hormones, including estrogen, progesterone, and androgens, are steroid hormones with a substantial impact on the immune system. Estrogen demonstrates both pro- and anti-inflammatory roles while progesterone and androgens are mainly anti-inflammatory. In this Research Topic, Yang et al. organized a clinical cohort study containing both female and male, smoking early stage non-small cell lung cancer (NSCLC) patients and performed single-cell RNA sequencing to reveal the heterogeneity of Tumor-Associated Macrophages (TAMs) within the tumor microenvironment (TME) (Yang et al.). Their studies noticed a higher estrogen level and more activated TAMs in the TME from female NSCLC patients than that from male patients (Yang et al.). This result is probably not too surprising as sex hormones share a common biosynthetic pathway with glucocorticoids, a group of strong anti-inflammatory agents, which predicts interactions with consequences on the immune response.

External factors such as diet may modulate the immune system through sex hormones. Increased 17β-estradiol after papaya consumption correlated with increased percentages of B cells and decrease in natural killer cells in healthy females (2). These regular changes may influence responses of the individual to infection and cancers.

In addition, the sex chromosomes also harbor some immune-related genes. For example, certain members of toll-like receptors and some cytokine receptors genes involved in T-cell and B-cell activity are located on the X chromosome and specific inflammatory pathway genes are situated on the Y chromosome. These sex-based immunological differences contribute to variations in the incidence of immune-related disease including autoimmune and cardiovascular diseases. Clearly, variations are expected in immune-based therapies including ICIs. Indeed, another interesting result from the NSCLC cohort study by Yang et al. included in this Research Topic was that toll-like receptors, interferons, and inflammatory cytokines including but not limited to IL-1 and IL-12 were significantly up-regulated in female-originated TAMs in comparison to those from the TAMs of male patients. It is worth noting that all these immune molecules are known to be related to immune activating pathways (Yang et al.). Their study also demonstrated the complement gene C1QC has significantly higher expression in female TAMs, while the matrix remodeling relevant genes FN1 and SPP1 are highly expressed in male-originated TAMs. High expression of the above three genes in immune cells are associated with poor prognosis independently. Moreover, they found that female TAMs show stronger immunogenicity with higher interferon-producing and antigen-presenting ability, while male TAMs upregulate the PPARs and matrix remodeling associated pathways, thus are more inclined to be immunosuppressive (Yang et al.). Early studies on the efficacy of ICI suggest males respond better, achieving improved overall survival (OS) and progression-free survival (PFS) from therapy with PD-1/PD-L1 inhibitors in both melanoma and NSCLC. In this case, increased frequencies of PD-1 expressing CD4+ T cells among male patients over female patients may be a contributory factor.

In this Research Topic, Sun et al. performed immunohistochemical studies on immune cell tumor-infiltration and PD-L1 expression on a gastrointestinal stromal tumor (GIST) cohort and reported an interesting finding that KIT receptor tyrosine kinase and platelet-derived growth factor receptor alpha (PDGFRA) mutations in GIST patients show significant differences in sex and cell types (Sun et al.). There were more female GIST patients with PDGFRA mutation than male GIST patients and KIT- and, PDGFRA-mutant GISTs have less tumor-infiltrating CD8+ T cells as compared with wild type-mutant GISTs (Sun et al.). The study also demonstrated that CD8+ T cell infiltration as well as PD-L1 expression were independent protective factors affecting the relapse free survival (RFS) of GIST patients, with high CD8+ T cell infiltration and high expression of PD-L1 being correlated with improved RFS.

Aging impairs immunity with the diminishing production of new immune cells. Higher basal levels of inflammatory factors in the elderly promote inflammatory diseases in the cardiovascular system and neurodegenerative diseases, reducing the ability to fight cancers and infections. To address the associated questions, in this Research Topic, Yang et al. organized a unique NSCLC cohort containing patients over the age of 75, and investigated whether these elderly patients could benefit more from pembrolizumab immunotherapy plus chemotherapy, or chemotherapy as a monotherapy. They reported that elderly NSCLC patients can benefit from pembrolizumab plus chemotherapy, and female senior patients exhibited the best responses with longer overall survival (Yang et al.). Thus, age and sex may alter the intensity of immune responses, increasing or decreasing thresholds, which are crucial in deciding the final outcome in immune-based treatment modalities. Also, in this Research Topic, Wong et al. review differences in the clinical efficacy and toxicity of ICIs in elderly patients and demonstrate that ICIs appear to have comparable efficacy and toxicity for younger and older patients, although meta-analysis and retrospective data suggest that the magnitude of benefit may be smaller in those over the age of 75 and in the setting of anti-CTLA-4 ICIs, potentially due to underlying changes associated with immunosenescence (Wong et al.). Their review also highlighted that older adults aged ≥65 had increased rate of immunotherapy-related adverse events as compared to younger patients aged 18-64, with patients who are more likely to suffer fatal irAEs from the older group (median age 70). However, younger patients may suffer unique adverse effects including but not limited to ICI-related infertility and premature menopause(Wong et al.). According to Wong et al., pregnancy is also a factor to be considered when weighing risks versus benefits for ICI in younger female patients, with the US FDA listing some ICIs in various risk categories for pregnancy. The underlying mechanisms for all these observations are obviously of genuine interest for future studies.

Nevertheless, we may only be scratching the surface of these targets as the immune system is well known for its complexity and redundancy. New targets may extend to innate immunity as the cells are just as capable of fighting cancers and infections and respond under regulation and control. Ultimately, in our efforts to retrain the immune system to regain its protective ability against cancers and infections, it is worthwhile to consider the need to overcome age and sex factors. We thank all the authors and reviewers for their invaluable work, which made the publication of this Research Topic possible. We hope that this collection of articles will serve as a useful source of knowledge to those interested in how sex and age can affect ICI immunotherapies.

## Author Contributions

All authors listed have made direct intellectual contributions to the work, and approved it for its publication. WL and PC made a substantial contribution when drafting this editorial essay.

## Funding

This study has been supported by Tsinghua University Spring Breeze Fund, Center for Life Sciences, and Institute for Immunology at Tsinghua University. This work is supported by grants from Ministry of Science and Technology of China Grants (2021YFC2300500 and 2021YFC2302403), National Natural Science Foundation of China (32141004, 81825010, 81730043, 81621002). This study is also supported by the Ministry of Higher Education Fundamental Research Grant Scheme, Malaysia (FRGS/1/2019/SKK11/TAYLOR/01/1).

## Conflict of Interest

The authors declare that the research was conducted in the absence of any commercial or financial relationships that could be construed as a potential conflict of interest.

## Publisher’s Note

All claims expressed in this article are solely those of the authors and do not necessarily represent those of their affiliated organizations, or those of the publisher, the editors and the reviewers. Any product that may be evaluated in this article, or claim that may be made by its manufacturer, is not guaranteed or endorsed by the publisher.
